# Conversion from farmland to orchard or agroforestry improves soil carbon sequestration by enhancing microbial biological activity in Northwest China

**DOI:** 10.1371/journal.pone.0344008

**Published:** 2026-03-06

**Authors:** Weixia Wang, Daniel F. Petticord, Shanchao Zhao, Guang Yang, Yuwen Chen, Xuansheng Huang

**Affiliations:** 1 College of Forestry and Landscape Architecture, Xinjiang Agricultural University, Urumqi, China; 2 Key Laboratory of Forestry Ecology and Industrial Technology, Xinjiang Education Department, Urumqi, China; 3 Cornell University, Ithaca, New York, United States of America; 4 Natural Forest Protection Center of Uygur Autonomous Region of Xinjiang, Urumqi, Xinjiang, China; Agriculture and Forestry University, NEPAL

## Abstract

Land-use conversion from conventional farmland to orchard or agroforestry systems holds great potential for enhancing soil organic carbon (SOC) sequestration and microbial activity in arid regions. This study investigated the impacts of such transitions in Northwest China, utilizing a 15-year chronosequence across seven land-use patterns. Soil profiles (0–30 cm) were analyzed for SOC, nutrient availability, and microbial biomass carbon (MBC) and nitrogen (MBN). Results demonstrated that the 15-year-old jujube-wheat alley cropping system (15JW) achieved the highest accumulation of SOC, MBC, and MBN, particularly in the topsoil (0–10 cm). Structural equation modeling (SEM) further elucidated the mechanisms driving microbial dynamics, revealing that substrate quality and phosphorus availability were the predominant controls. Specifically, the model explained 47.4% of the variation in MBC (R^2^ = 0.474), with SOC exerting a significant direct influence (β = −0.35). In contrast, available phosphorus (AP) was identified as the primary driver of MBN (β = 0.52), contributing to 56.1% of its total variance (R^2^ = 0.561). These findings suggest that long-term agroforestry management mitigates carbon loss by fostering stable nutrient-mediated microbial pools. Our research underscores that transitioning to mature agroforestry systems is a strategic measure for improving soil fertility and climate resilience in semi-arid ecosystems.

## Introduction

Human land‐use practices are the single greatest threat to soil quality in the Anthropocene [1]. This is especially true in arid and semi-arid regions, where shifts in land cover associated with different land-use types drives environmental change and soil degradation [2]. These land-use changes often lead to declining soil quality, often represented in reductions to soil organic matter, with consequences for the physical, chemical, and biological properties of soil [3–6]. Recent evidence further suggests that land-use intensity remains a major driver of soil microbial community composition and soil carbon dynamics across diverse ecosystems [7,8].

Evaluating soil quality requires careful consideration of key biological and chemical indicators [9]. In particular, soil microorganisms – which mediate approximately 90% of organic-matter decomposition – are both engines of soil nutrient cycles and extremely sensitive to global change, meaning microbes may represent reliable indicators of soil health [10]. Although microbial biomass carbon (MBC) and microbial biomass nitrogen (MBN) contribute only 1–3% of total soil C and 1–5% of total soil N, the role of microbes in soil formation, development and nutrient cycling is critical [10]. The cycling of carbon (C), nitrogen (N), and phosphorus (P) in soil is fundamentally linked to microbial activity [11].

Microbial biomass thus serves as both a “sink” and a “source” of soil C and N, playing an essential role in forest and other ecosystems’ carbon and nitrogen cycles [12,13]. Because microbial biomass is relatively sensitive to local environmental changes, shifts in microbial biomass are widely used as a proxy to evaluate soil quality [14]. Indicators such as MBC and the ratio of MBC to total organic carbon (TOC) provide valuable insights into soil health, particularly in assessing the impacts of anthropogenic disturbances [15,16]. For example, recently, Parajuli *et al* [17]. demonstrated that different land-use practices strongly modify microbial biomass with knock-on consequences for organic carbon cycling, reinforcing the diagnostic value of these microbial indicators in diverse management systems.

Thus, understanding how MBC and MBN respond to land-use change, and how they relate to soil quality and fertility, is critical for promoting sustainable management of forest and agro-ecosystems. Numerous studies have explored the dynamics of soil C and microbial properties in the context of land conversion, particularly from natural vegetation to cropland or grazed pastures [3,18–20]. However, two key gaps remain. First, most prior work has focused on conversion from natural vegetation to cropland or pasture; far fewer studies have examined transitions from conventional farmland to orchard systems or agroforestry [7,8]. Second, there is a need for a more mechanistic framework that links land-use conversion → changes in organic-matter input + nutrient availability → microbial biomass responses → soil quality outcomes [7]. Recent reviews highlight microbial carbon-use efficiency (CUE) and microbial community functional shifts under land-use change, underscoring the importance of integrating microbial traits into predictive models of soil C dynamics [6,7].

In the semi-arid region of Northwest China – one of the country’s largest fruit-producing areas [21,22] – agricultural restructuring has led to widespread conversion of farmland into orchards or intercropping systems. Such transitions hold considerable socio-economic promise but may substantially alter the soil organic carbon (SOC) pool and microbial biomass, thereby influencing soil quality, especially in soils already low in organic C. Recent meta-analyses have confirmed that agroforestry systems can enhance soil carbon sequestration, particularly in arid regions, by stimulating plant carbon inputs and microbial carbon turnover [7,8]. Nevertheless, the specific microbial-biomass responses to farmland → orchard or agroforestry conversion in this region remain underexplored, particularly regarding the mediating roles of nutrient availability and organic-matter inputs. Although our preliminary study [23] established the efficacy of labile organic carbon fractions as descriptive indicators, the current work moves beyond these metrics to elucidate the underlying microbial-mediated mechanisms. Specifically, we examine how MBC and MBN respond to nutrient stoichiometry, providing a functional perspective on soil carbon sequestration that was not addressed in our previous investigation.

In this study, we (1) explicitly focus on the conversion from conventional farmland to jujube orchard or alley-cropping systems in a semi-arid, low-SOC environment; (2) quantify MBC and MBN dynamics along the soil profile to link microbial indicators with nutrient supply and vertical distribution patterns; (3) employ structural equation modeling (SEM) to identify the primary soil physicochemical controls (e.g., SOC, TN, P, bulk density (BD)) on microbial biomass, thereby providing a mechanistic understanding of how land-use change affects microbial pools and soil quality; and (4) assess the potential of agroforestry systems in semi-arid lands to enhance soil quality and contribute to climate-resilient land management.

Therefore, the specific objectives of this study are as follows: (1) To quantify changes in SOC, MBC, and MBN distribution throughout the soil profile across three land-use types. (2) To identify the primary soil physicochemical factors controlling MBC and MBN using SEM-based path analysis. (3) To evaluate whether agroforestry conversion may enhance soil microbial biomass and SOC in a semi-arid, low-SOC environment, thereby supporting sustainable land management and climate resilience.

The conceptual framework of this study is illustrated in the Graphical Abstract (S1 Fig).

## Materials and methods

### Study area

This study was conducted in Wensu County, Xinjiang Province, northwestern China (40°52′-42°21′N, 79°28′-81°30′E), which has a warm-temperate arid climate. The region receives an average annual precipitation of only 65.4 mm against a high potential evaporation exceeding 1,800 mm. Soils are classified as sandy loam, and the area serves as a major hub for fruit production, particularly jujube (*Ziziphus jujuba*). In recent years, agricultural restructuring has promoted the conversion of farmland to jujube orchards and alley-cropping systems, providing an ideal setting to investigate the effects of land-use change on soil quality and microbial dynamics.

To assess the temporal dynamics of this land-use change, we adopted a chronosequence approach (space-for-time substitution). We selected a series of sites representing three distinct systems: wheat monoculture (control), jujube-wheat alley-cropping, and pure jujube orchards. To ensure the validity of this spatial comparison, all plots were located within geographical proximity to maintain consistent soil parent materials, topography, and microclimate. Notably, all jujube-based systems were converted from homogeneous wheat croplands 5, 10, and 15 years prior to the study, while adjacent continuous wheat fields served as the baseline. All plots followed a standardized management regime, including five irrigations and four fertilizations annually, synchronized with key phenological stages (seedling, flowering, fruit-setting, and fruit expansion). Fertilization was uniform across sites, utilizing farmyard manure, diammonium phosphate, and urea.

Seven land-use management practices were examined (hereafter referred to as treatments):

(1) wheat monoculture field (*Mono*), (2) 5-year-old jujube orchard (*5J*), (3) 5-year-old jujube–wheat alley-cropping system (*5JW*), (4) 10-year-old jujube orchard (*10J*), (5) 10-year-old jujube–wheat alley-cropping system (*10JW*), (6) 15-year-old jujube orchard (*15J*), and (7) 15-year-old jujube–wheat alley-cropping system (*15JW*). All the orchards we selected are privately owned. We obtained permission from the farm owners before entering the orchards to collect soil samples.

### Soil sampling

In each land-use type, three distinct orchards were selected as experimental replicates. Soil samples were collected using a 5-cm diameter auger at 10-cm intervals from the soil surface to 30 cm depth at the end of August. In each orchard, six jujube trees were randomly selected, with sampling sites positioned within one meter of the tree crown projection, but away from the trunk. Samples from the same soil layer within each orchard were combined to form a composite sample. For the alley cropping systems, six jujube trees were randomly selected with sampling sites similarly placed within one meter of the tree crown projection. In the intercropped areas, six additional sampling sites were arranged in an S-shaped pattern within the crop rows. Samples from each soil layer in the alley cropping system were mixed into a composite sample. In total, 9 soil samples were taken from each land-use type (3 distinct orchards, 3 soil depths). For the farmland sites, six samples were arranged in an S-shaped pattern, and sampling was performed in the same manner as in the orchards. A total of 9 soil samples were collected across 3 farmland areas. For BD analysis, soil core samples were collected from the 0–30 cm layers using a 100-cm³ BD corer. Field-moist soil samples were passed through a 2-mm sieve and divided into two subsamples. One subsample was placed in cloth bags and stored at 4°C for microbial biomass analysis. The other subsample was air-dried and stored at room temperature for subsequent chemical analyses.

### Soil physiochemical analysis

Soil water content and BD were measured gravimetrically by drying soil samples at 105°C for at least 48 hours. SOC was analyzed using the K₂Cr₂O₇-H₂SO₄ calefaction method [24], while total nitrogen (TN) was measured using the semimicro-Kjeldahl digestion method, followed by analysis with a flow injection auto-analyzer (FIA Lachat Instruments USA). Total potassium (TK) was measured using a nitric acid-perchloric acid (HNO₃-HClO₄) deboiling method, and total phosphorus (TP) was determined with the sulfuric acid-perchloric acid (H₂SO₄-HClO₄) deboiling method. Ammonium nitrogen (AN) was measured using indophenol blue colorimetry. Available potassium (AK) was determined using a flame photometer, and available phosphorus (AP) was measured through a diacid (HCl-H₂SO₄) extraction method.

### Soil microbial biomass carbon and nitrogen

Soil MBC and MBN were determined using the chloroform fumigation-extraction method [25]. This method is based on the difference between carbon or nitrogen extracted from fumigated and non-fumigated soil samples, using 0.5 M K₂SO₄ as the extracting agent. Soil samples from each site were divided into paired 20 g subsamples. One subsample was immediately extracted with 60 ml of 0.5 M K₂SO₄, while the second subsample was fumigated with chloroform vapor for 48 hours in a desiccator, followed by 10 vacuum/purge cycles, and then extracted as described above. The extractable organic C and TN in the K₂SO₄ extracts, both before and after fumigation, were measured using a total C/N analyzer (Multi-N/C 2100®, Analytik Jena AG, Germany). The released C and N were converted to MBC and MBN using established conversion factors. MBC was calculated as:


$$\text{MBC }=\;E_{C}\;/\;K_{EC}$$ (1)

Where *E*_C_ = (organic C extracted from fumigated soils) – (organic C extracted from non-fumigated soils), and *K*_EC_ = 0.38 [25].


$$\text{MBN }=\;E_{N}\;/\;K_{EN}$$ (2)

Where *E*_N_ = (N in fumigated soils) - (N in non-fumigated soils), and *K*_EN_= 0.45.

### Statistical analyses

Analysis of variance (ANOVA) was used to evaluate differences in soil microbial properties, including MBC, MBN, the MBC to TOC ratio, and the MBC ratio, as well as SOC content across the seven land-use types and soil depths. The significance of the effects of land-use type, soil depth, and their interactions on microbial properties was assessed. Least significant difference (LSD) analysis was applied to compare means, with a significance level set at P < 0.05. Pearson correlation analysis was used to determine the correlation coefficients between different soil nutrient indicators and MBC and MBN. All statistical analyses were conducted using SPSS version 25.0 and SigmaPlot version 10.0 (SPSS Inc Chicago IL USA).

SEM is a comprehensive multivariate statistical approach that analyzes complex causal relationships on the basis of the covariance matrix of variables [26]. Its core advantage is that it integrates path analysis and factor analysis while simultaneously accounting for measurement error and the structural relationships among latent variables [27]. In this study, we aimed to identify the key drivers of soil MBC and MBN in the study region. We constructed SEMs with MBC and MBN as response variables, implemented in R (version 4.4.1) using the lavaan package. Path specifications were iteratively refined, and overall model fit was evaluated using χ²/df, the goodness of fit index (GFI), the standardized root mean square residual (SRMR), and the root mean square error of approximation (RMSEA). Model fit was considered good and acceptable when 1 < χ²/df < 3, SRMR < 0.08, GFI > 0.90, and RMSEA < 0.06.

### Inclusivity in global research

Additional information regarding the ethical, cultural, and scientific considerations specific to inclusivity in global research is included in S2 File.

## Results

### Soil nutrient analysis

During the initial transition from farmland to orchard intercropping, TN content first decreased, then increased as the ecosystem recovered, and orchard vegetation established (Table 1). In the 15JW treatment, TN content was significantly higher than in the farmland plots (P < 0.05). The patterns for AN and AK followed a similar trend, with both showing an initial decline and subsequent increase as restoration advanced. Overall, soil nutrient content in the 0–30 cm layer increased between 1.78% and 161.80% over time, depending on the treatment and restoration stage. Overall, available nutrient concentrations (AN, AP, and AK) showed higher responsiveness than total nutrients, with AP exhibiting the most substantial increase of 161.8% in the subsoil (20–30 cm) of 15JW compared to the monoculture (Table 1).

**Table 1:** Effect of land use type on soil nutrients.

Index	Soil depth (cm)	Mono	5J	5JW	10J	10JW	15J	15JW
TN (g/kg)	0-10	1.04 ± 0.0a^a^	1.10 ± 0.02b^a^	0.95 ± 0.01c^a^	1.15 ± 0.02d^a^	1.07 ± 0.02ab^a^	1.25 ± 0.03e^a^	1.19 ± 0.01d^a^
	10-20	0.89 ± 0.06a^b^	0.97 ± 0.01ac^b^	0.90 ± 0.02a^a^	1.12 ± 0.01bde^a^	0.99 ± 0.03c^b^	1.19 ± 0.01d^b^	1.10 ± 0.01e^b^
	20-30	0.75 ± 0.02a^c^	0.86 ± 0.02b^c^	0.68 ± 0.01c^b^	0.95 ± 0.01d^b^	0.77 ± 0.01a^c^	1.04 ± 0.01e^c^	0.90 ± 0.03b^c^
TP (g/kg)	0-10	1.12 ± 0.10a^a^	1.14 ± 0.01a^a^	1.15 ± 0.02a^a^	1.19 ± 0.08a^ab^	1.79 ± 0.21b^a^	1.88 ± 0.09b^a^	2.25 ± 0.06c^a^
	10-20	1.07 ± 0.11a^a^	1.09 ± 0.01a^a^	1.12 ± 0.03a^a^	1.32 ± 0.03ab^a^	1.42 ± 0.09b^ab^	1.45 ± 0.13b^ab^	1.82 ± 0.08c^b^
	20-30	0.99 ± 0.05a^a^	0.97 ± 0.04a^b^	1.03 ± 0.05a^a^	1.10 ± 0.04ac^b^	1.15 ± 0.18ac^b^	1.35 ± 0.16bc^b^	1.18 ± 0.05ac^c^
TK (g/kg)	0-10	21.46 ± 2.41a^a^	23.87 ± 0.40ab^a^	23.46 ± 0.40ac^a^	25.85 ± 0.40bcd^a^	26.59 ± 0.03be^a^	26.6 ± 0.02bf^a^	28.18 ± 0.40def^a^
	10-20	19.08 ± 2.11a^a^	21.08 ± 0.40ab^b^	22.64 ± 0.41bc^a^	24.66 ± 0.79 cd^ab^	25.46 ± 0.00d^b^	25.46 ± 0.00d^b^	25.81 ± 0.39d^b^
	20-30	18.69 ± 2.43a^a^	20.26 ± 0.38ab^b^	21.86 ± 0.68abd^a^	22.66 ± 1.05bc^b^	23.45 ± 0.40bc^c^	25.41 ± 0.00c^c^	24.90 ± 0.35 cd^b^
AN (mg/kg)	0-10	126.13 ± 11.93ac^a^	123.1 ± 3.44a^a^	127.1 ± 2.88ac^a^	160.49 ± 19.64bd^a^	157.00 ± 7.39 cd^a^	200.45 ± 9.01e^a^	216.57 ± 10.67e^a^
	10-20	108.13 ± 5.46ab^a^	94.37 ± 5.31a^b^	114.15 ± 2.42bc^b^	127 ± 6.27ce^ab^	148.97 ± 2.29df^a^	144.63 ± 9.95ef^b^	162.37 ± 6.59f^b^
	20-30	100.2 ± 10.06a^a^	89.62 ± 5.07a^b^	107.73 ± 2.17ab^b^	106.06 ± 8.48ab^b^	125.84 ± 7.96bc^b^	132.49 ± 5.65c^b^	144.54 ± 9.03c^b^
AP (mg/kg)	0-10	76.50 ± 8.91a^a^	90.39 ± 1.43a^a^	110.75 ± 2.76b^a^	110.51 ± 3.86b^a^	136.52 ± 1.45c^a^	144.20 ± 6.70c^a^	144.20 ± 6.21c^a^
	10-20	60.44 ± 3.84a^a^	63.22 ± 0.82a^b^	56.23 ± 2.54a^b^	79.09 ± 2.20b^b^	78.35 ± 5.14b^b^	96.05 ± 1.43c^b^	109.81 ± 4.78d^b^
	20-30	34.98 ± 5.05a^b^	48.74 ± 0.49b^c^	48.06 ± 1.01b^c^	62.99 ± 3.92c^c^	69.86 ± 4.03c^b^	86.63 ± 0.71d^b^	91.58 ± 4.33d^c^
AK (mg/kg)	0-10	370.13 ± 52.79ab^a^	316.59 ± 3.46a^a^	388.23 ± 5.89b^a^	422.92 ± 13.06bc^a^	487.02 ± 9.97 cd^a^	551.13 ± 19.95de^a^	569.98 ± 6.53e^a^
	10-20	302.25 ± 39.91a^a^	311.31 ± 1.51a^a^	377.67 ± 6.53b^ab^	385.21 ± 19.95b^a^	431.21 ± 4.20bc^b^	475.71 ± 20.99c^b^	460.62 ± 15.08c^b^
	20-30	287.17 ± 34.56a^a^	262.89 ± 4.13a^b^	356.55 ± 12.07b^b^	377.67 ± 26.13bc^a^	347.50 ± 15.08b^c^	413.72 ± 5.23 cd^c^	434.23 ± 6.53d^b^

Values represent the mean of three replicates. Lower case letters in each line: significant differences (P < 0.05) at different land use patterns in the same soil depth. Superscript letters in each column: significant differences (P < 0.05) at different soil depths in the same land use pattern.

### Soil organic carbon content

Land use type and soil depth had significant individual effects on SOC content, although their interaction was not statistically significant (Table 2). Overall, the impact of land use on SOC was more pronounced in the topsoil (0–10 cm) than in the deeper layers (Table 3).

**Table 2:** ANOVA table of *F*-values on the effect of land use type and soil depth on soil properties (SOC, MBC, MBN, MBC/MBN and MBC/SOC).

Variable	Land use type		Soil depth		Interaction	
	F	P	F	P	F	P
SOC	11.173	< 0.001	29.744	< 0.001	0.89	0.564
MBC	3.284	0.01	17.817	< 0.001	0.479	0.916
MBN	4.249	0.002	25.703	< 0.001	0.4	0.956
MBC/MBN	0.469	0.827	0.487	0.618	0.082	1.000
MBC/SOC	0.542	0.773	5.68	0.007	0.147	1.000
df	6		2		12	

**Table 3:** Effect of land use type on soil microbial biomass carbon content soil microbial biomass nitrogen content.

Index	Soil depth (cm)	Mono	5J	5JW	10J	10JW	15J	15JW
SOC(g/kg)	0-10	5.74 ± 0.09a^a^	8.09 ± 0.13b^a^	8.80 ± 1.05bc^a^	9.94 ± 0.43bc^a^	10.30 ± 0.69bc^a^	9.56 ± 1.40bc^a^	10.59 ± 0.19c^a^
	10-20	5.45 ± 0.11a^a^	5.69 ± 0.28a^b^	7.42 ± 1.05ab^ab^	7.07 ± 0.34ab^b^	8.84 ± 0.27b^a^	8.88 ± 1.21b^a^	8.76 ± 0.82b^ab^
	20-30	4.95 ± 0.08ab^b^	4.58 ± 0.46a^c^	5.70 ± 0.44ac^b^	5.93 ± 0.46ac^b^	6.64 ± 0.41bce^b^	7.97 ± 1.22de^a^	7.03 ± 0.88ce^b^
MBC/SOC	0-10	3.24 ± 0.15a^a^	3.50 ± 0.40a^a^	3.45 ± 0.72a^a^	3.35 ± 0.24a^a^	3.43 ± 0.33a^a^	3.4 ± 1.38a^a^	4.05 ± 1.02a^a^
	10-20	2.24 ± 0.16a^b^	3.35 ± 0.41a^a^	2.76 ± 0.32a^a^	3.24 ± 0.63a^a^	2.68 ± 0.11a^ab^	3.04 ± 1.36a^a^	2.97 ± 0.64a^a^
	20-30	1.74 ± 0.12a^c^	2.1 ± 0.14a^b^	2.2 ± 0.34a^a^	2.21 ± 0.32a^a^	2.12 ± 0.46a^b^	2.74 ± 1.41a^a^	2.91 ± 0.26a^a^
MBC/MBN	0-10	9.8 ± 1.62a^a^	9.61 ± 1.28a^a^	9.16 ± 2.48a^a^	8.03 ± 0.59a^a^	7.83 ± 0.67a^a^	10.14 ± 4.41a^a^	7.43 ± 0.33a^a^
	10-20	10.16 ± 1.36a^a^	10.73 ± 0.60a^a^	7.91 ± 1.38a^a^	10.31 ± 3.22a^a^	8.74 ± 0.68a^a^	10.20 ± 4.93a^a^	7.99 ± 1.12a^a^
	20-30	10.50 ± 1.66a^a^	10.55 ± 0.65a^a^	9.55 ± 0.67a^a^	9.79 ± 0.41a^a^	10.69 ± 4.11a^a^	11.15 ± 3.97a^a^	8.37 ± 2.51a^a^

Values represent the mean of three replicates. Lower case letters in each line: significant differences (P < 0.05) at different land use patterns in the same soil depth.

Superscript letters in each column: significant differences (P < 0.05) at different soil depths in the same land use pattern.

Following the transition from farmland to orchard or agroforestry systems, topsoil SOC levels were significantly elevated across all treatments compared to the monoculture. SOC accumulation increased with stand age, with the most substantial gains observed in the 15-year intercropping system (15JW). Specifically, topsoil SOC in the 15JW treatment reached 10.59 g/kg, representing an 84.5% increase relative to the monoculture (from 5.74 to 10.59 g/kg, Table 3). Notable increases were also recorded in the 10JW (79.4%, 10.30 g/kg), 10J (73.2%, 9.94 g/kg), 15J (66.6%, 9.56 g/kg), 5JW (53.3%, 8.80 g/kg), and 5J (40.9%, 8.09 g/kg) treatments (Table 3). These results indicate that agroforestry intercropping and increased stand age synergistically promote carbon sequestration in the surface soil layer.

### Soil microbial biomass C and N

**Fig 1:**
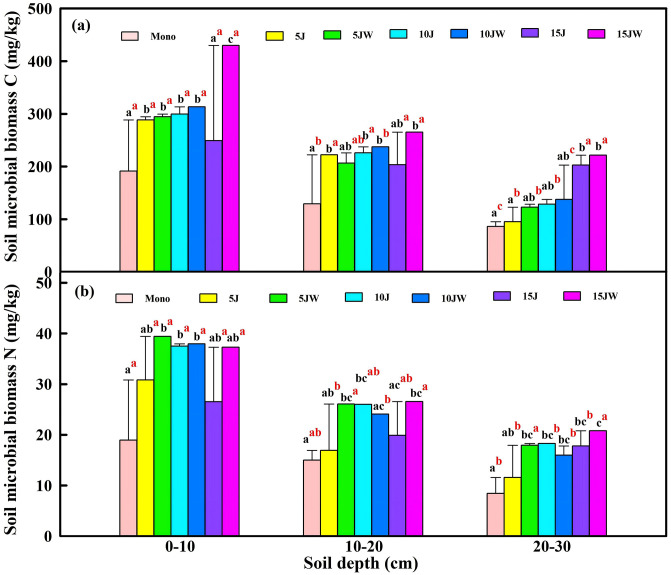
Effect of land use type on (a) soil microbial biomass carbon content, (b) soil microbial biomass nitrogen content. Values represent the mean of three replicates. Lower case letters: significant differences (P < 0.05) at different land use patterns in the same soil depth. Superscript letters: significant differences (P < 0.05) at different soil depths in the same land use pattern.

Soil MBC varied significantly with land-use conversion to orchard or agroforestry systems. MBC ranged from 86.2 to 429.8 mg/kg, with a mean value of 295.0 mg/kg in the topsoil layer, compared to 142.1 mg/kg in the lower soil layer (Fig 1a). This variation was primarily driven by the elevated MBC observed in the 15JW treatment (Fig 1a), while the lowest MBC was recorded in the Mono treatment. In the topsoil, MBC followed the order: 15JW > 10JW > 10J > 5JW > 5J > 15J > Mono (Fig 1a).

Soil MBN exhibited a similar pattern, ranging from 8.4 to 39.4 mg/kg, with significant variation across treatments. In the topsoil, MBN followed the order: 5JW > 10JW > 10J > 15JW > 5J > 15J > Mono (Fig 1b). Both MBC and MBN were significantly influenced by land use and soil depth, but no significant interaction between land use and depth was observed (Table 2).

Across all land-use transitions and soil depths, no statistically significant effects of land-cover change were found on the ratio of MBC to MBN (Table 3). However, the ratio of MBC to SOC (mean 3.5%) was slightly but significantly higher in the topsoil (0–10 cm) compared to the bottom layer (20–30 cm), where the ratio averaged 2.3% (Table 3). Soil depth had a significant effect on the MBC to SOC ratio (Table 2).

**Fig 2:**
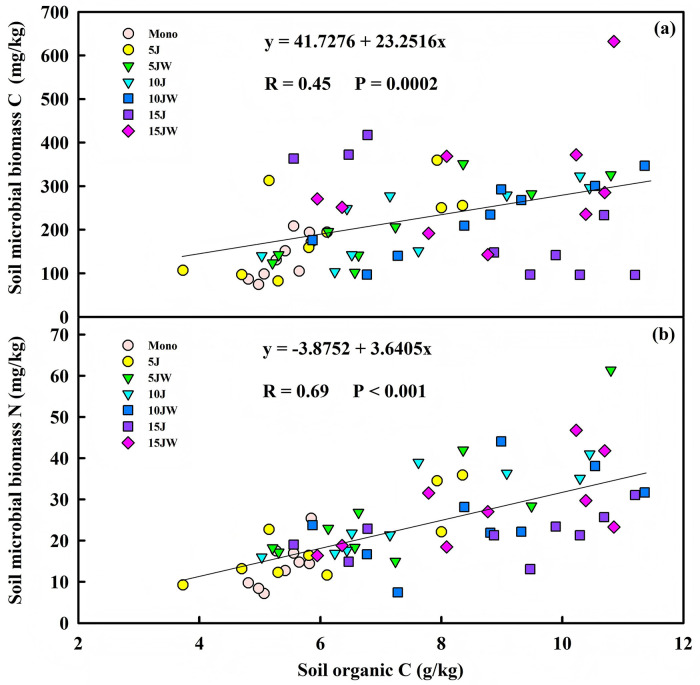
Relationship between soil organic carbon and (a) soil microbial biomass carbon, (b) soil microbial biomass nitrogen.

**Fig 3:**
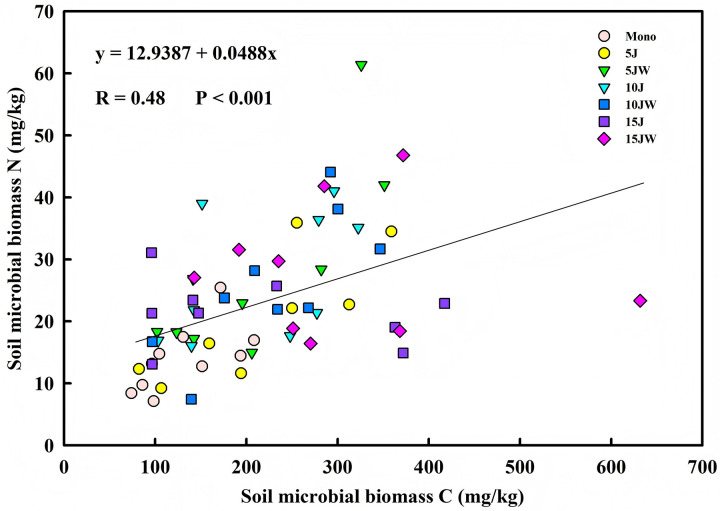
Relationship between soil microbial biomass carbon and soil microbial biomass nitrogen.

Linear regression analyses revealed a significant correlation between SOC and soil MBC (R = 0.45, n = 63, P = 0.0002), as well as between SOC and MBN (R = 0.69, n = 63, P < 0.001) (Fig 2). Additionally, MBC was significantly correlated with MBN (R = 0.48, n = 63, P < 0.001) (Fig 3).

### Correlation and structural equation model analysis

To elucidate the relationships between soil nutrients and MBC, MBN under varying land-use patterns in Wensu County, Aksu, a pearson correlation analysis was conducted. The results demonstrated that the conversion of farmland into jujube orchards or jujube-wheat intercropping systems established significant positive correlations (P < 0.05) between soil nutrient indicators and microbial biomass parameters.

**Fig 4:**
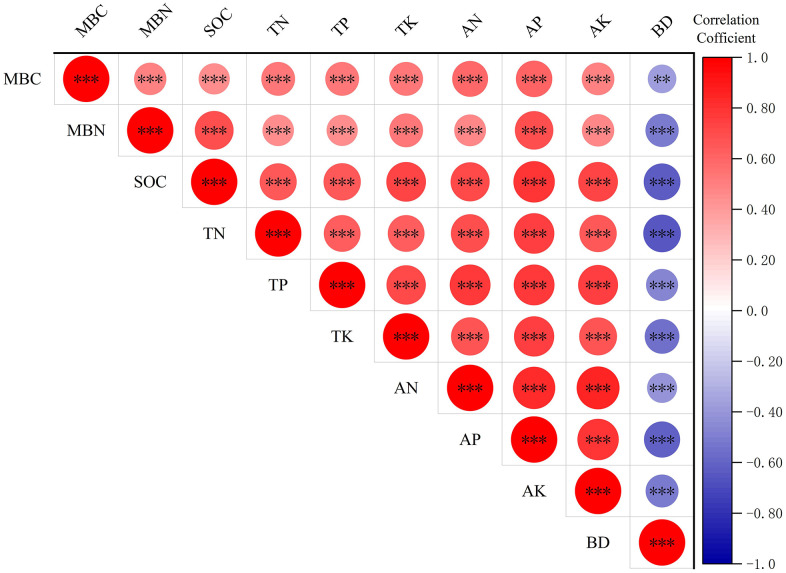
Correlation analysis of soil nutrients and soil microbial biomass carbon, soil microbial biomass nitrogen. Note: *** indicates p < 0.001. ** indicates p < 0.01. * indicates p < 0.05. The color depth and circle size reflect the size of the correlation coefficient.

Specifically, MBC and MBN were both significantly and positively correlated with total nutrients (TN, TP, and TK) and available nutrients (AN, AP, and AK) (Fig 4). These robust correlations suggest that the accumulation of soil nutrients during land-use transition directly supports the expansion of the microbial pool.

**Fig 5:**
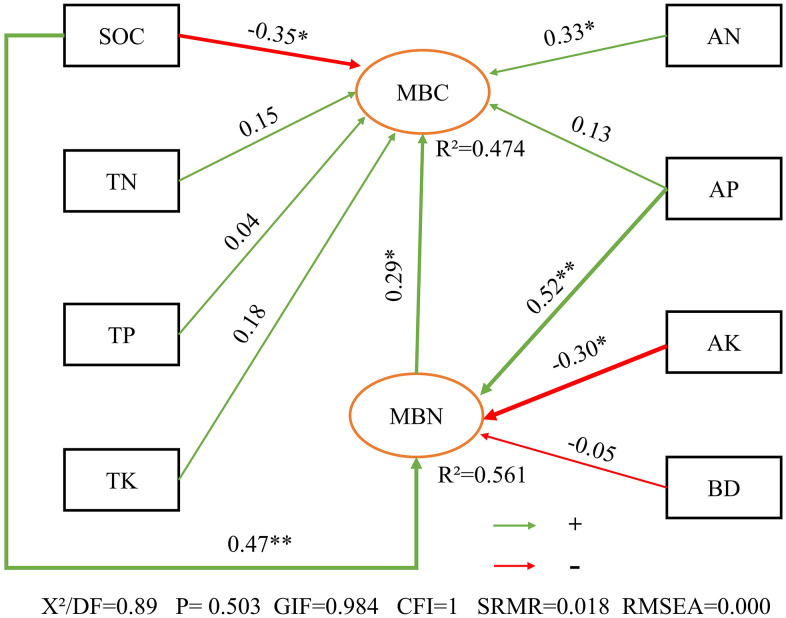
Pathway analysis of influencing factors of soil microbial biomass carbon and soil microbial biomass nitrogen. Note:Arrows represent hypothesized causal directions, with the numbers next to the arrows indicating the strength of standardized path coefficients. Green arrows indicate positive relationships, while red arrows denote negative relationships. Only statistically significant relationships are shown (* P < 0.05, ** P < 0.01, *** P < 0.001). The percentage below the ellipses represents the proportion of variance in MBC and MBN explained by the model. Variance Explanation: The percentage below the ellipses indicates the proportion of variance in MBC and MBN explained by the model.

To further disentangle the direct and indirect drivers of microbial dynamics, SEM was constructed (Fig 5). The SEM fit indices indicated an excellent correspondence between the model and the experimental data: χ²/df = 0.89, GFI = 0.984, CFI = 1.000, SRMR = 0.018, and RMSEA = 0.000.

Path analysis revealed distinct regulatory mechanisms for MBC and MBN. The combined factors of SOC, TN, TP, TK, AN, AP, and MBN explained 47.4% of the total variation in MBC (R^2^ = 0.474). Notably, SOC exerted the strongest direct influence on MBC, characterized by a negative path coefficient (β = −0.35), suggesting a potential trade-off between carbon sequestration and immediate microbial accessibility. This was followed by the significant positive effects of AN (β = 0.33), MBN (β = 0.29), and TK (β = 0.18). Other variables, including TN (β = 0.15), AP (β = 0.13), and TP (β = 0.04), showed minor but detectable impacts, indicating that MBC dynamics are collectively shaped by both substrate quality and nutrient availability.

In contrast, SOC, AP, AK, and BD accounted for 56.1% of the variation in MBN (R^2^ = 0.561). AP was identified as the predominant driver of MBN (β = 0.52), followed by SOC (β = 0.47). Conversely, AK (β = −0.30) and BD (β = −0.05) exhibited negative direct influences on MBN. These findings underscore the critical role of phosphorus availability in modulating microbial nitrogen immobilization in arid agroecosystems.

The underlying raw data for soil properties and microbial activities are provided in S3 File.

## Discussion

### Soil microbial biomass C and N

Transitioning from farmland to orchard or agroforestry systems markedly increased MBC and MBN, particularly under the 15JW intercropping system (125.6%) when comparing to the monoculture setting. With increasing stand age, stable reserves of soil organic matter begin to accumulate. Previous work suggests greater SOC availability stimulates microbial growth and activity [9,28,29], which would be consistent with our results (see below, *Soil Organic Carbon Content*). There was a clear increase in soil MBN when comparing the monoculture to the orchard and agroforestry settings, with the exception of the 5 year jujube orchard (Fig 1). The increase in MBN is likely due to an increase in microbial activity associated with increased organic matter inputs and a reduction in physical disturbance to the SOC pool – as discussed below monoculture wheat systems are regularly plowed. Tilling disrupts the community and may be responsible for the reduction in MBN. In deeper soil layers, a more pronounced increase in MBN with time became more obvious, particularly in the 15-year JW system. This correlated with a commensurate increase in MBC, and is probably a symptom of a stable and active microbial community that is regularly interacting with increasingly-deep rooted plants in the older setting. This strong dependency of MBN on AP suggests that AP acts as a metabolic ‘gatekeeper’ for nitrogen assimilation in these arid alkaline soils. According to the stoichiometric theory of [30], phosphorus is a prerequisite for the synthesis of P-rich ribosomal RNA, which in turn drives protein synthesis and microbial N immobilization. Thus, the relief of P-limitation during land-use conversion likely facilitated the coupling of microbial C and N uptake.

A relatively constant MBC/MBN ratio across treatments indicates stable microbial stoichiometry. This stability reflects the stoichiometric homeostasis of the soil microbial community [30]. Even under varying resource supplies, microbial populations can buffer external imbalances by adjusting their internal nutrient allocation or community composition to maintain fundamental physiological requirements. This homeostatic behavior suggests that the microbial biomass composition in our study sites is constrained by highly conserved metabolic traits regardless of management history. The MBC/SOC ratio (1.7–4.0%) falls within the reported range [15] but was slightly higher in intercropping systems (3.6%) than in monocultures (3.4%), implying more efficient substrate utilization and microbial turnover due to diversified root exudates and organic inputs. These responses are particularly meaningful in the nutrient-poor soils of Wensu County (SOC ≈ 1%), where MBC and MBN serve as sensitive indicators of soil quality and nutrient cycling intensity.

In our SEM analysis, the direct path from SOC to MBC was negative (β = −0.35), contrasting with their positive zero-order correlation. This discrepancy suggests that the direct influence of SOC on microbial biomass is suppressed when accounting for the covariance of soil N, P, and BD. Ecologically, this negative direct association likely reflects a shift in SOC quality; as jujube orchards mature, a larger proportion of carbon is sequestered in stable fractions that are less accessible to microbes [31]. Thus, while the accumulation of recalcitrant SOC is associated with reduced microbial carbon use efficiency, the overall positive trend observed in the field is likely driven by indirect, nutrient-mediated pathways [32]. Integrating simple correlations with SEM outcomes reveals a nuanced trade-off land-use conversion indirectly promotes microbial growth by improving the nutrient environment, even as the ‘quality’ of the primary carbon substrate declines.

This apparent ‘decoupling’ between total SOC quantity and microbial biomass expansion can be elucidated through the lens of soil structure and carbon quality. As orchards mature, reduced tillage promotes the formation of macro-aggregates, which physically sequester organic carbon and restrict its accessibility to microbes [33]. Furthermore, the shift in substrate quality aligns with the Microbial Efficiency-Matrix Stabilization (MEMS) framework [34]. This model posits that high-input, recalcitrant litter in perennial systems predominantly contributes to stable soil organic matter through physico-chemical stabilization rather than fueling immediate microbial biomass growth.

### Soil organic carbon content

Land-use conversion had a high impact on SOC accumulation, particularly in the 0–10 cm topsoil layer—an observation consistent with Ferreira *et al* [3]. Compared with cropland, both orchard and agroforestry systems exhibited significantly elevated SOC levels. Older orchards had higher SOC we may infer is related to greater carbon accumulation stemming from long-term inputs from tree litter, root turnover, and reduced tillage disturbance. Agricultural plowing accelerates SOC mineralization by disrupting aggregates and enhancing oxygen diffusion [35], whereas orchard soils are untilled and consequently more stable – minimizing decomposition and promoting carbon stabilization [36,37].

Increased SOC may also increase rates of nitrogen cycling, as relaxing carbon limitation increases microbial activity, and microbes may release inorganic nitrogen (NH₄⁺ and NO₃⁻) through mineralization, shifting N into the MBN pool [38–40]. This stimulatory effect of rising SOC on MBC and MBN is consistent with our observations that microbial biomass rose under orchard and intercropping systems. One unexplored contribution may be the proliferation of earthworms in less disturbed soil. Earthworms may also contribute to the formation of stable aggregates that protect SOC from decomposition [41]. Such structural stabilization, coupled with microclimatic moderation (e.g., lower temperature and moisture fluctuations), fosters long-term SOC sequestration [42,43]. Moreover, the interaction between plants and microbes is further modulated by priming effects [44]. While stable carbon is sequestered, the continuous input of fresh root exudates in agroforestry systems may stimulate specific microbial guilds to process existing soil organic matter, creating a dynamic equilibrium between carbon stabilization and nutrient mineralization. SOC accumulation typically follows a saturation trajectory, gradually reaching a new equilibrium where carbon inputs balance decomposition losses [45]. Conversion from cropland to orchard soils may require several decades (≈ 40 years) to stabilize [37,46], though the timescale varies with management intensity, vegetation type, and climatic conditions [47,48]. It does appear, in this case, that as orchards mature and root systems deepen, sustained organic inputs and moderated microclimates could produce increased accumulation of SOC [36,49].

Interestingly, while MBC increased after the conversion from farmland to orchard or agroforestry systems, the MBC/SOC ratio remained unchanged or decreased. This pattern likely arises because SOC accumulated concurrently or even more rapidly than microbial biomass, particularly in older orchard soils with enhanced litter input and root turnover [50,51]. Our findings suggest that the unchanged or slightly reduced MBC/SOC ratio does not contradict enhanced microbial activity or carbon sequestration but rather indicates a shift toward more stable carbon accumulation and long-term improvement in soil carbon storage efficiency [52]. Consequently, our current results extend our previous indicator-based conclusions [23] by providing a more nuanced mechanistic explanation; we reveal that the long-term evolution of soil quality in these arid agroecosystems is fundamentally driven by microbial-mediated nutrient cycling and is constrained by both carbon quality and phosphorus availability.

While agroforestry effectively enhances soil carbon sequestration and microbial biomass, it should not be viewed as a stand-alone solution for carbon mitigation. The net climate benefit of such systems depends on broader agricultural emissions, including those from fertilizer use, irrigation, and soil management. Therefore, integrating agroforestry with optimized nutrient and water management practices is essential to maximize mitigation potential. A systems-based approach that considers all greenhouse gas sources and sinks within agricultural landscapes will provide a more realistic assessment of sustainable carbon management strategies.

## Conclusions

Our study demonstrates that converting farmland to orchard and agroforestry systems significantly enhances SOC and nutrient sequestration in arid regions. The 15-year jujube-wheat intercropping system (15JW) represents the most effective model for improving soil quality, with benefits most pronounced in the 0–10 cm topsoil. Beyond simple accumulation, our findings reveal that AP acts as a critical metabolic “gatekeeper” regulating microbial nitrogen immobilization, whereas the direct influence of SOC on microbial biomass is constrained by substrate quality and physical sequestration within macro-aggregates. Despite these dynamic shifts, the soil microbial community maintains consistent stoichiometric homeostasis, ensuring stable MBC:MBN ratios across management types. While land-use conversion remains a potent climate mitigation strategy, SOC accumulation follows a clear saturation trajectory over time. Future management must therefore transition from merely increasing inputs to optimizing nutrient-mediated microbial processes to balance long-term carbon stabilization with sustained nutrient cycling.

## Supporting information

S1 Fig Graphical abstract. (A visual summary of the conversion from farmland to orchard/agroforestry and its impact on soil carbon sequestration.).
(PPTX)

S2 File Inclusivity in global research checklist. (A completed questionnaire outlining the ethical and collaborative considerations for the research conducted in Northwest China.).
(DOCX)

S3 File Dataset. (Original data including soil organic carbon, microbial biomass carbon, microbial biomass nitrogen, and other relevant soil physicochemical properties.).
(XLS)

## Abbreviations

Mono: wheat monoculture field
5J: 5-year-old jujube orchard
10J: 10-year-old jujube orchard
15J: 15-year-old jujube orchard
5JW: 5-year-old jujube/wheat alley cropping system
10JW: 10-year-old jujube/wheat alley cropping system
15JW: 15-year-old jujube/wheat alley cropping system
SOC: soil organic carbon
TOC: total organic carbon
CUE: carbon-use efficiency
MBC: microbial biomass carbon
MBN: microbial biomass nitrogen
SMB: soil microbial biomass
C: carbon
N: nitrogen
P: phosphorus
TN: total nitrogen
TK: total potassium
TP: total phosphorus
AN: ammonium nitrogen
AK: available potassium
AP: available phosphorus
SMB: soil microbial biomass
SEM: structural equation model
BD: bulk density
