# Supplementary material for: Conversion from farmland to orchard or agroforestry improves soil carbon sequestration by enhancing microbial biological activity in Northwest China
Source: PLoS One. 2026 Mar 6;21(3):e0344008. doi: 10.1371/journal.pone.0344008 (PMC12965561; doi:10.1371/journal.pone.0344008)
Supplement: S1 Fig — (A visual summary of the conversion from farmland to orchard/agroforestry and its impact on soil carbon sequestration.). (PPTX) [file pone.0344008.s001.pptx]

## Slide 1
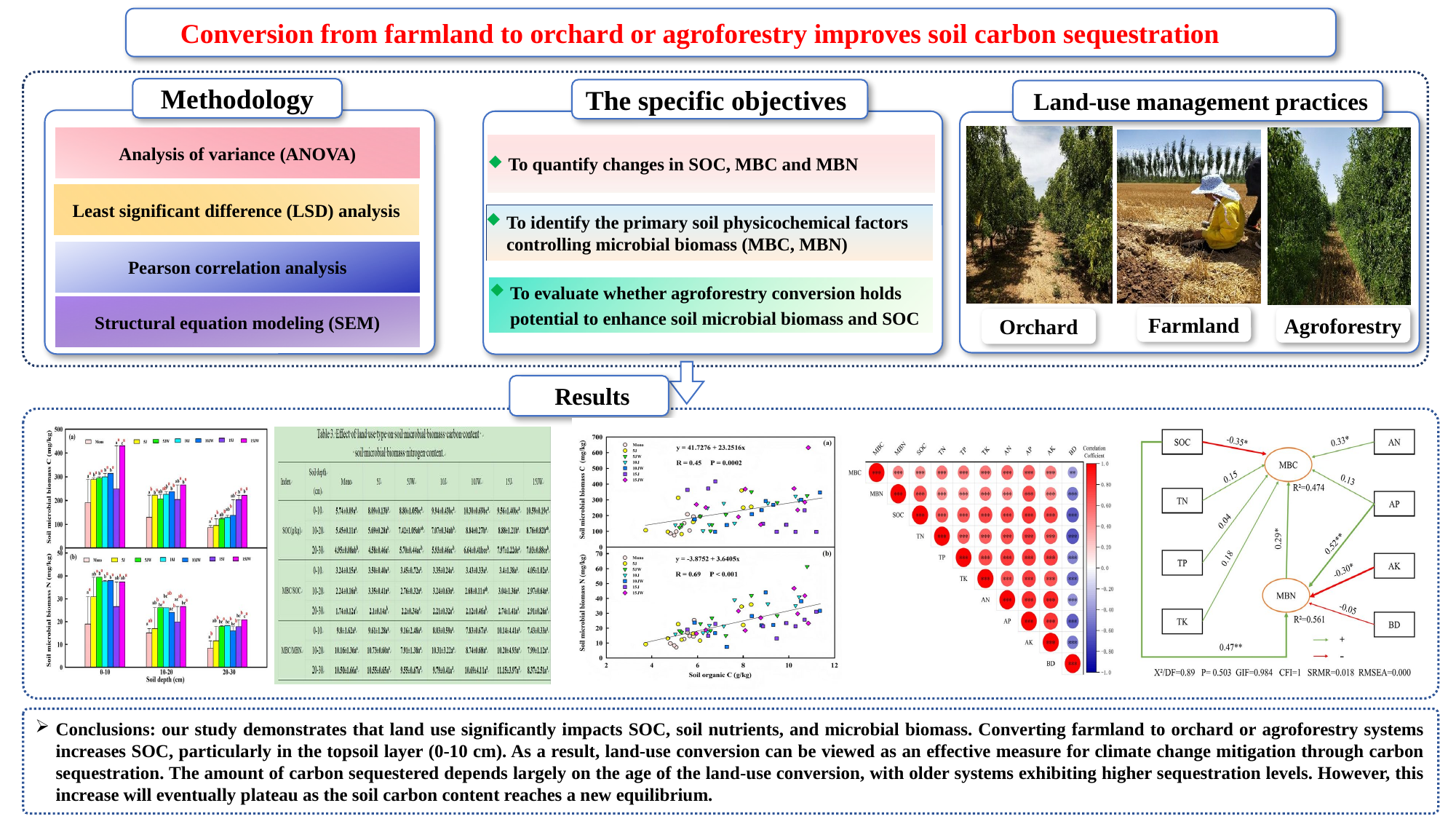

Conversion from farmland to orchard or agroforestry improves soil carbon sequestrationChina
Methodology
The specific objectives
 Land-use management practices
Analysis of variance (ANOVA)
To quantify changes in SOC, MBC and MBN
Least significant difference (LSD) analysis
To identify the primary soil physicochemical factors controlling microbial biomass (MBC, MBN)
Pearson correlation analysis
To evaluate whether agroforestry conversion holds potential to enhance soil microbial biomass and SOC
Structural equation modeling (SEM)
Farmland
Agroforestry
Orchard
 Results
Conclusions: our study demonstrates that land use significantly impacts SOC, soil nutrients, and microbial biomass. Converting farmland to orchard or agroforestry systems increases SOC, particularly in the topsoil layer (0-10 cm). As a result, land-use conversion can be viewed as an effective measure for climate change mitigation through carbon sequestration. The amount of carbon sequestered depends largely on the age of the land-use conversion, with older systems exhibiting higher sequestration levels. However, this increase will eventually plateau as the soil carbon content reaches a new equilibrium.
